# Comparative study of four immortalized human brain capillary endothelial cell lines, hCMEC/D3, hBMEC, TY10, and BB19, and optimization of culture conditions, for an *in vitro* blood–brain barrier model for drug permeability studies

**DOI:** 10.1186/2045-8118-10-33

**Published:** 2013-11-22

**Authors:** Daniela E Eigenmann, Gongda Xue, Kwang S Kim, Ashlee V Moses, Matthias Hamburger, Mouhssin Oufir

**Affiliations:** 1 Pharmaceutical Biology, Department of Pharmaceutical Sciences, University of Basel, Klingelbergstrasse 50, 4056 Basel, Switzerland; 2 Mechanisms of Cancer, Friedrich Miescher Institute for Biomedical Research, Maulbeerstrasse 66, 4058 Basel, Switzerland; 3 Department of Pediatrics, John Hopkins University, 200 N. Wolfe Street, Baltimore, MD 21287, USA; 4 Vaccine and Gene Therapy Institute, Oregon Health and Science University (OHSU), 505 NW 185th Ave, Beaverton, OR 97006, USA

**Keywords:** Endothelial cell line, *In vitro* human blood–brain barrier (BBB) model, 24-well tissue culture insert, Transendothelial electrical resistance (TEER), Paracellular permeability, CellZscope

## Abstract

**Background:**

Reliable human *in vitro* blood–brain barrier (BBB) models suitable for high-throughput screening are urgently needed in early drug discovery and development for assessing the ability of promising bioactive compounds to overcome the BBB. To establish an improved human *in vitro* BBB model, we compared four currently available and well characterized immortalized human brain capillary endothelial cell lines, hCMEC/D3, hBMEC, TY10, and BB19, with respect to barrier tightness and paracellular permeability. Co-culture systems using immortalized human astrocytes (SVG-A cell line) and immortalized human pericytes (HBPCT cell line) were designed with the aim of positively influencing barrier tightness.

**Methods:**

Tight junction (TJ) formation was assessed by transendothelial electrical resistance (TEER) measurements using a conventional epithelial voltohmmeter (EVOM) and an automated CellZscope system which records TEER and cell layer capacitance (C_CL_) in real-time.

Paracellular permeability was assessed using two fluorescent marker compounds with low BBB penetration (sodium fluorescein (Na-F) and lucifer yellow (LY)). Conditions were optimized for each endothelial cell line by screening a series of 24-well tissue culture inserts from different providers. For hBMEC cells, further optimization was carried out by varying coating material, coating procedure, cell seeding density, and growth media composition. Biochemical characterization of cell type-specific transmembrane adherens junction protein VE-cadherin and of TJ proteins ZO-1 and claudin-5 were carried out for each endothelial cell line. In addition, immunostaining for ZO-1 in hBMEC cell line was performed.

**Results:**

The four cell lines all expressed the endothelial cell type-specific adherens junction protein VE-cadherin. The TJ protein ZO-1 was expressed in hCMEC/D3 and in hBMEC cells. ZO-1 expression could be confirmed in hBMEC cells by immunocytochemical staining. Claudin-5 expression was detected in hCMEC/D3, TY10, and at a very low level in hBMEC cells. Highest TEER values and lowest paracellular permeability for Na-F and LY were obtained with mono-cultures of hBMEC cell line when cultivated on 24-well tissue culture inserts from Greiner Bio-one® (transparent PET membrane, 3.0 μm pore size). In co-culture models with SVG-A and HBPCT cells, no increase of TEER could be observed, suggesting that none of the investigated endothelial cell lines responded positively to stimuli from immortalized astrocytic or pericytic cells.

**Conclusions:**

Under the conditions examined in our experiments, hBMEC proved to be the most suitable human cell line for an *in vitro* BBB model concerning barrier tightness in a 24-well mono-culture system intended for higher throughput. This BBB model is being validated with several compounds (known to cross or not to cross the BBB), and will potentially be selected for the assessment of BBB permeation of bioactive natural products.

## Background

Endothelial microvascular capillary cells in the human brain constitute a unique cellular barrier to sustain brain homeostasis and to protect the brain from xenobiotics and neurotoxic metabolites circulating in the bloodstream. It has been estimated that more than 98% of small-molecule drugs are not able to cross the blood–brain barrier (BBB) [1]. Hence, BBB penetration is a major challenge in the development of drugs acting on the central nervous system (CNS), where penetration into the brain is pivotal for achieving therapeutic effects [2]. On the other hand, low CNS penetration is desirable for drugs acting in the periphery. In early drug discovery and development, new chemical entities (NCEs) are now screened for their ability to cross the BBB.

For this purpose, a wide range of *in silico*, *in vitro*, and *in vivo* BBB models for early prediction of brain permeability of compounds have been developed and established [3]. Computational models and physicochemical methods such as the Parallel Artificial Membrane Permeability Assay (PAMPA-BBB) offer high-throughput screening capability at early stages of drug discovery, but are only able to predict passive permeation [4,5]. In contrast, *in vivo* models such as *in situ* brain perfusion provide high-quality data and some of the most reliable measurements of BBB drug penetration [6]. However, they are expensive in terms of time and resources and, therefore, only suitable for testing of compounds at more advanced stages of development [7]. Cell-based *in vitro* BBB models using primary or immortalized brain capillary endothelial cells from animal or human origin cultivated on microporous filter membranes of Transwell systems may bridge the gap between *in silico* and *in vivo* studies. They have been used for *in vitro* drug BBB permeability assessment for a long time, and their simple design allows for cost-efficient high-throughput screening [8-10].

Since mono-culture systems represent a highly simplified model and are far from mimicking *in vivo* conditions, further brain-derived cells being part of the neurovascular unit, such as astrocytes, pericytes, and/or neurons, have been incorporated into double and triple co-culture *in vitro* BBB models [10]. Whereas astrocytes have repeatedly been shown to favorably influence barrier tightness of endothelial cells [11-14], the impact of pericytes on BBB models is still a matter of controversy. It was shown that a syngeneic tri-culture model with primary rat brain capillary endothelial cells, astrocytes, and pericytes yielded highest transendothelial electrical resistance (TEER) values [15]. In contrast, another study showed that a barrier strengthening effect of pericytes critically depended on the differentiation state of cells [16].

*In vitro* animal BBB models using primary or low passage porcine, bovine, rat, or mouse cells, partly in double and triple co-culture systems, are generally characterized by relatively high TEER values and by low paracellular permeability of marker compounds [17]. Despite these favorable features, *in vitro* animal models show major drawbacks. Isolation and purification procedures of primary cells are tedious and time-consuming and require substantial experience [9]. Yields and lifespan of isolated cells are limited, and animal endothelial cells show differences in the expression of drug transporters and efflux pumps when compared to human brain capillary endothelial cells [18]. The use of primary cultures from human origin would avoid interspecies pharmacogenetic variation, yet the availability of these cells is greatly limited on ethical grounds [19]. Immortalized human brain capillary endothelial cells can be used as an alternative. These cells proliferate indefinitely and preserve their differentiating properties after repetitive passages, which is desirable for standardized screenings [9]. However, the establishment of a reliable human cell-line based BBB model has proven to be difficult [17]. These cells typically form only limited restrictive monolayers *in vitro,* with TEER values in the range 20 to 200 Ωcm^2^[17,20,21]. Compared to *in vivo* conditions where TEER values have been estimated to exceed 1000 Ωcm^2^[22,23], this is considerably lower. Despite this limitation*, in vitro* models with human immortalized cell lines possess several advantages and may be favorable tools for obtaining first mechanistic insights into BBB permeability of drugs. However, optimization of *in vitro* human BBB models for best barrier tightness is a prerequisite.

This study provides comparative data on four known immortalized human brain capillary endothelial cell lines, hCMEC/D3 [24], hBMEC [25], TY10 [26], and BB19 [27], regarding their ability to form a restrictive barrier in an *in vitro* 24-well format BBB model intended for higher throughput drug permeability screening. For the first time, immortalized human astrocytes (SVG-A cell line [28]) and immortalized human pericytes (HBPCT cell line [29]) were included into co-culture models with these endothelial cell lines, with the objective to increase barrier tightness. We also present here a large set of *in vitro* TEER data recorded for each endothelial cell line cultivated on a range of tissue culture inserts from different manufacturers, with the aid of a CellZscope on-line TEER recording system [30]. This information was important prior to optimizing an *in vitro* BBB model with a particular cell line, since material and pore size of the filter membrane of the tissue culture inserts have been reported to strongly affect the adherence of cells and barrier tightness [31,32]. Subsequent optimization of a model with hBMEC cells was done by systematically screening various coating materials and coating procedures, by testing a variety of growth media containing barrier-strengthening compounds, by replacing the commonly used fetal bovine serum (FBS) with human serum (HS), and by using astrocyte-conditioned medium (ACM). In addition to TEER measurements, paracellular permeability of two fluorescent tracer molecules that do not cross the BBB in a significant amount (sodium fluorescein (Na-F) and lucifer yellow (LY)) was assessed. Biochemical characterization of VE-cadherin, ZO-1, and claudin-5, three major components of adherens and tight endothelial junctions, was carried out for each endothelial cell line. Furthermore, we performed immunostaining for ZO-1 in hBMEC cells.

## Methods

### Chemicals and materials

NaCl, CaCl_2_, MgCl_2_, KCl, glucose, 4-(2-hydroxyethyl)piperazine-1-ethanesulfonic acid (HEPES), NaHCO_3_, Dulbecco’s modified Eagle medium (DMEM), human collagen type IV, fibronectin, hydrocortisone (HC), dexamethasone, human epidermal growth factor (hEGF), 8-(4-chlorophenylthio) adenosine-3’ ,5’-cyclic monophosphate sodium salt (8-(4-CPT)cAMP), 4-(3-butoxy-4-methoxybenzyl) imidazolidin-2-one (RO-20-1724), sodium fluorescein (Na-F), radio-immunoprecipitation assay (RIPA) lysis buffer, phosphate buffered saline (PBS), Triton X-100, bovine serum albumin (BSA), and 4’ ,6-diamidino-2-phenylindole dihydrochloride (DAPI) were purchased from Sigma-Aldrich (Steinheim, Germany). Fetal bovine serum (FBS) “Gold” was from PAA Laboratories (Pasching, Austria). Human AB serum (HS) from a healthy female donor was obtained from the blood donor bank (Blutspendezentrum Universität Basel, Switzerland). Both sera were heat inactivated for 30 min at 56°C before use. EBM-2 and Single-Quots (human vascular endothelial growth factor, insulin-like growth factor-1, human fibroblast growth factor-B, hEGF, ascorbic acid, heparin, and HC) were from Lonza (Basel, Switzerland). Lucifer Yellow VS dilithium salt (LY) was purchased from Santa Cruz (Heidelberg, Germany). Antibiotic-antimycotic solution, secondary antibody (Alexa Fluor 488 conjugation), and phalloidin (Alexa Fluor 647) were received from Life Technologies (Paisley, UK). Rat-tail collagen type I and matrigel were purchased from BD Biosciences (Bedford, USA) and from Trevigen (Gaithersburg, MD, USA). The antibodies specific for zonula occludens (ZO)-1 protein, claudin-5, and vascular endothelial (VE)-cadherin were from Abcam (Cambridge, UK). Protease inhibitor cocktail was from Roche (Basel, Switzerland). Tissue culture flasks were from BD Biosciences (Bedford, USA) and from TPP (Trasadingen, Switzerland). 24-well tissue culture inserts and 24-well plates were from Corning Incorporated® (Corning, NY, USA), Greiner Bio-one® (Frickenhausen, Germany), BD Falcon® (Le Pont de Claix, France), Millipore® (Billerica, MA, USA), Nunc® (Roskilde, Denmark), and Brand® (Wertheim, Germany).

### Cell cultures

**Figure 1:**
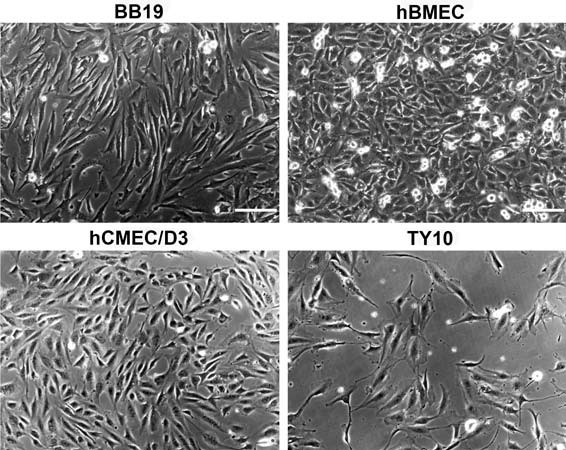
**Phase contrast microscopy of four immortalized human brain capillary endothelial cell lines.** Four endothelial cell lines were cultured in growth medium containing 20% FBS for 2 days and the representative cell morphology was imaged with a phase contrast microscope. Scale bar: 150 μm.

In this study, four immortalized human brain capillary endothelial cell lines, hCMEC/D3 [24], hBMEC [25], TY10 [26], and BB19 [27], were used (Figure 1). Immortalized hCMEC/D3 cell line was kindly provided by Prof. Pierre-Olivier Couraud (Institut Cochin, Université René Descartes, Paris, France). hBMEC cell line was obtained from Prof. Kwang Sik Kim and Prof. Dennis Grab (John Hopkins University, Baltimore, MD, USA) through the Swiss Tropical and Public Health Institute (Prof. Reto Brun, STPH, Basel, Switzerland). TY10 cell line, a new generation of conditionally immortalized cells coming from TY08 [33], which was established by the same method as TY09 cell line [34], was obtained from Prof. Takashi Kanda (Yamaguchi University Graduate School of Medicine, Ube, Yamaguchi, Japan). BB19 cells were kindly provided by Prof. Ashlee V. Moses (Oregon Health and Science University, Portland, OR, USA). The immortalized SVG-A cell line, an astrocytic cell subclone of the astroglial SVG cell line [28,35], was a generous gift from Prof. Avindra Nath (National Institute of Neurological Disorders and Stroke, Bethesda, MD, USA) and the immortalized human pericyte cell line HBPCT [29] was provided by Prof. Takashi Kanda (Yamaguchi University Graduate School of Medicine, Ube, Yamaguchi, Japan).

All cell lines (except TY10) were cultured and grown to confluence in rat-tail collagen type I coated tissue culture flasks at 37°C and 5% CO_2_ in humid atmosphere. TY10 cells were cultured under similar conditions but at 33°C, since this cell line was immortalized with a hTERT and a temperature-sensitive SV40 large-T antigen allowing the cells to grow at the permissive temperature of 33°C and to differentiate into physiological endothelial cells at the non-permissive temperature of 37°C [26,33,34]. TY10, hBMEC, and BB19 cells were cultured in EBM-2 supplemented with Single-Quots, antibiotic-antimycotic solution, and 20% FBS (growth medium 20% FBS). hCMEC/D3 cells were cultured either in growth medium containing 20% FBS, or in the initial culture medium containing 5% FBS as described previously [24]. Culture medium for SVG-A and HBPCT cells was DMEM supplemented with antibiotic-antimycotic solution and 10% FBS. For experiments, hCMEC/D3 cells were between passage (P) 25 and 32, hBMEC cells were between P12 and P25, TY10 cells between P10 and P19, BB19 cells between P11 and P19, SVG-A cells between P7 and P10, and HBPCT cells between P13 and P19. All endothelial cell lines used in this study have been reported to preserve their phenotype for a limited range of passages (for hCMEC/D3: up to P33 [24], for hBMEC: up to P25, for TY10: at least up to P50 [34], and for BB19: up to P21 [27]).

### Biochemical and immunocytochemical characterization of cellular junctions

Examined cells were lysed in standard RIPA lysis buffer supplied with protease inhibitor cocktail for 30 min on ice. Cleared supernatant corresponding to 50 μg of total protein per sample was subjected to SDS-PAGE and western blotting analysis. For immunochemistry studies, hBMEC cells grown on coverslips coated with rat-tail collagen type I were fixed with paraformaldehyde for 20 min at room temperature and subsequently permeabilized with PBS containing 0.2% Triton X-100 for 10 min. The cells were then blocked with 3% BSA for 30 min and incubated with ZO-1 antibody (1:100) at 4°C overnight. Secondary antibody (Alexa Fluor 488 conjugation) was incubated together with phalloidin (Alexa Fluor 647) for 1 h and the representative images were taken using a fluorescent microscope (Zeiss Z1). The nuclei were stained with DAPI.

### *In vitro* co-culture BBB models and TEER measurements

Contact and non-contact co-culture BBB models using immortalized brain capillary endothelial cells and immortalized astrocytes or immortalized pericytes were established as follows. hCMEC/D3 (grown with initial medium), hBMEC, TY10, and BB19 cells were seeded separately on the apical side of the filter membranes of 24-well tissue culture inserts from Corning® (polycarbonate (PC) and polyester (PES) membranes, 0.4 μm and 3.0 μm pore size), coated with rat-tail collagen type I (10 μg/cm^2^). The cell seeding density varied between 3.0 × 10^4^ and 15 × 10^4^ cells/cm^2^ (hCMEC/D3: 6.0 × 10^4^ cells/cm^2^, hBMEC: between 3.0 × 10^4^ and 4.5 × 10^4^ cells/cm^2^, TY10: 3.0 × 10^4^ cells/cm^2^, and BB19: between 7.5 × 10^4^ and 15 × 10^4^ cells/cm^2^). Beforehand, SVG-A or HBPCT cells were seeded on the collagen type I coated (10 μg/cm^2^) basal side of the porous filter membrane (SVG-A: between 3.0 × 10^4^ and 9.0 × 10^4^ cells/cm^2^, HBPCT: 3.0 × 10^4^ cells/cm^2^) and allowed to attach for 1 h. For non-contact models, SVG-A cells were seeded at a cell density of 1.6 × 10^4^ cells/cm^2^ onto the culture plate and incubation thereafter was 2 h (37°C, 5% CO_2_). After the start of the experiment*,* TEER values were measured manually every 2–3 days using an epithelial voltohmmeter (EVOM) coupled to an Endohm-6 measurement chamber (World Precision Instruments, USA). Each TEER reading was followed by an exchange of medium. TEER values for cell layers, expressed in Ωcm^2^, were calculated by subtracting the resistance of a coated control insert without cells from a coated insert with cells and by subsequent correction for surface area. For each experiment, at least 2 replicates were measured. Results are expressed as means ± S.E.M.

### Screening of 24-well inserts from different providers for mono-culture BBB models

For the screening of 24-well tissue culture inserts from different providers (Corning®, Greiner Bio-One®, BD Falcon®, Millipore®, Nunc®, and Brand®), each endothelial cell line (hCMEC/D3 (cultured with growth medium 20% FBS), hBMEC, TY10, and BB19) was seeded separately on the apical side of the filter membrane at a density varying between 1.5 × 10^4^ and 17 × 10^4^ cells/cm^2^. Prior to seeding, the membranes were coated with rat-tail collagen type I. The tissue culture inserts were placed into a 24-well cell module of a CellZscope system (NanoAnalytics, Münster, Germany) [30] which was placed inside an incubator (37°C and 5% CO_2_). For *in vitro* models with TY10 cells, further experiments were performed for which the CellZscope system was first placed at 33°C (5% CO_2_) for 2 days and subsequently transferred to 37°C (5% CO_2_). The medium was refreshed every 2–4 days. TEER values were recorded in real-time every hour. TEER values for cell layers, expressed in Ωcm^2^, were obtained by subtracting the TEER of a coated control insert without cells from a coated insert with cells. After placing the CellZscope system into the incubator, the cell module needs at least 6 h to reach 37°C. Since TEER values are highly temperature sensitive [36], recorded TEER values in this time period were not considered to be valid and are not reported. For each *in vitro* experiment, 2 or 3 replicates were measured. Results are expressed as means ± S.E.M. In addition to TEER values, the CellZscope system monitors the cell layer capacitance (C_CL_) which reflects the membrane surface area. C_CL_ values in the range of 0.5-5.0 μF/cm^2^ indicate cell confluency and validate TEER values [37,38]. All reported TEER values in the result section belong to a C_CL_ within this range, if not reported otherwise.

### Optimization of mono-culture *in vitro* BBB models

For further optimization of mono-culture systems with hBMEC, various insert coating procedures using matrigel (80 μg/cm^2^) and a mixture of collagen type IV/fibronectin (80/20 μg/cm^2^) were assessed. Several growth media were tested containing compounds such as HC (500 nM; 1500 nM; in addition to HC already included in Single-Quots), dexamethasone (500 nM; 1000 nM), hEGF (50 ng/mL; 100 ng/mL; in addition to hEGF already included in Single-Quots), 8-(4-CPT)cAMP (250 μM), and RO-20-1724 (17.5 μM), which have been used in BBB studies to induce barrier tightness [17,21,39,40]. To avoid cytotoxic effects of DMSO or ethanol, their final concentration in the growth medium was below 0.25% [41]. Moreover, a culture medium (EBM-2, Single-Quots, and antibiotic-antimycotic solution) called “HS medium” supplemented with HS (10% and 20%, respectively) instead of FBS was evaluated. Astrocyte-conditioned media (ACM) were collected from SVG-A cells cultured with either growth medium for endothelial cell lines (ACM-1) or culture medium for SVG-A (ACM-2) and stored at -20°C until use.

### BBB permeability studies with Na-F and LY

**Figure 2:**
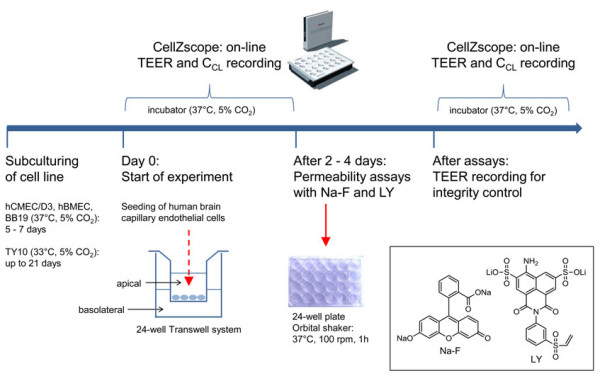
**Scheme of preparation of the *in vitro *human BBB model.** After subculturing the human cell lines for several days, the brain capillary endothelial cells were seeded on microporous filter membranes of 24-well tissue culture inserts (Greiner Bio-one®, transparent PET membrane, 3.0 μm pore size). The inserts were immediately transferred to a 24-well cell module of a CellZscope system which was placed inside an incubator (37°C and 5% CO_2_). TEER and C_CL_ values were recorded on-line every hour. After 2–4 days (at the maximal TEER), paracellular permeability of Na-F and LY was assessed. To monitor barrier integrity after the assay, TEER and C_CL_ values were again recorded real-time using the CellZscope system.

**Figure 3:**
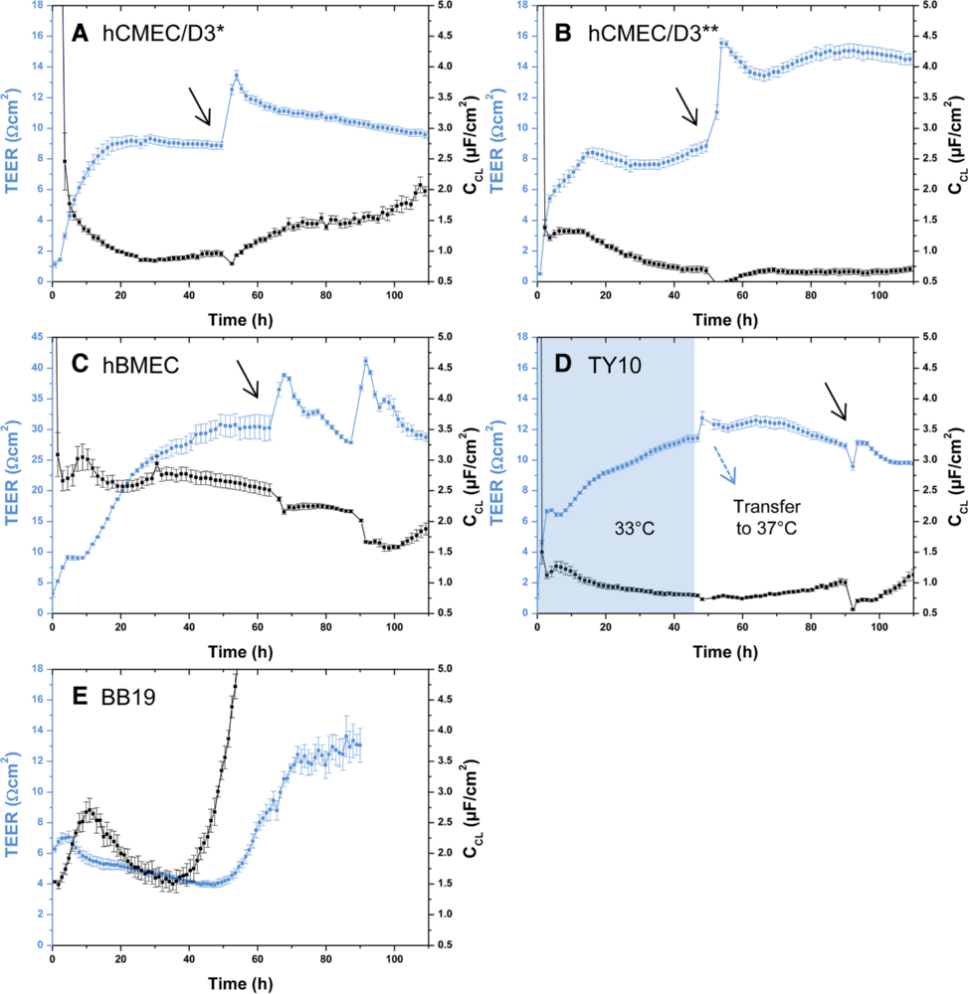
**Mean TEER values (blue curve) and C**_**CL **_**values (black curve) recorded real-time by the CellZscope system of four human brain capillary endothelial cell lines grown on 24-well tissue culture inserts.** Black arrows indicate the time when the permeability assay with Na-F was performed (for resulting P_app_ values see Figures 7A and B). For hCMEC/D3 and TY10, the permeability assay was performed at TEER values in the range of 10 Ωcm^2^ (hCMEC/D3: *cultured with initial medium **(A)**, **cultured with growth medium 20% FBS **(B)**. For hBMEC, the assay was carried out at TEER values in the range of 30 Ωcm^2^. TEER values after the assay were in the same range or even higher as before, suggesting that cell layers were robust during the experiment and barrier integrity was maintained **(A-D)**. For each of the three cell lines, C_CL_ values were in the acceptable range of 0.5-5.0 μF/cm^2^, indicating that cells were confluent. BB19 cell line **(E)** yielded very low TEER values in the range of 5 Ωcm^2^. Since C_CL_ values were drastically increasing after 55 h, the experiment was stopped. BB19 cells were not included into permeability studies with Na-F and LY due to their low TEER. All experiments were performed with Greiner Bio-one® inserts (transparent PET membrane, 3.0 μm pore size, 0.6 × 10^6^ pores/cm^2^, n = 2–5).

To further assess tight junction (TJ) integrity of endothelial cell layers, paracellular permeabilities of Na-F (MW 376.27) and LY (MW 550.39) were measured. These two fluorescent, non-P-glycoprotein (P-gp) substrate molecules have low BBB penetration and are widely used as barrier integrity markers for *in vitro* models. To obtain comparative data, hCMEC/D3 cells, hBMEC cells, and TY10 cells were seeded onto 24-well tissue culture inserts (Greiner Bio-one®, transparent polyethylene terephthalate (PET) membrane, 3.0 μm pore size, 0.6 × 10^6^ pores/cm^2^) and incubated at 37°C (5% CO_2_) (except TY10 cells) inside the CellZscope cell module (Figure 2). For TY10, the CellZscope system was first put at 33°C (5% CO_2_) for 2 days and was thereafter transferred to 37°C (5% CO_2_) for 2 days prior to the permeability assay (Figure 3D). Cell seeding density for hCMEC/D3 and hBMEC was 6.0 × 10^4^ cells/cm^2^, and for TY10 it was 3.0 × 10^4^ cells/cm^2^. For hBMEC, contact co-culture models with SVG-A cells (9.0 × 10^4^ cells/cm^2^) or HBPCT cells (6.0 × 10^4^ cells/cm^2^) on the basal side of the coated filter membranes were tested.

TEER and C_CL_ values before and after the assays were monitored continuously for integrity control. All experiments for Na-F permeability were carried out at the time indicated with a black arrow in Figures 3A-D. For LY permeability assessment, TEER values were in the same range (graphs not shown). BB19 cells were not included in this permeability study since TEER values were extremely low.

For the permeability assay, tissue culture inserts were transferred into a 24-well plate containing 700 μL of pre-warmed (37°C) Ringer HEPES buffer (150 mM NaCl, 2.2 mM CaCl_2_, 0.2 mM MgCl_2_, 5.2 mM KCl, 2.8 mM glucose, 5 mM HEPES, and 6 mM NaHCO_3_, pH 7.4) in each well (basolateral compartment). Medium in inserts (apical compartment) was then replaced with 300 μL of a pre-warmed (37°C) working solution containing Na-F or LY at 10 μg/mL in Ringer HEPES buffer. The 24-well plate was incubated at 37°C on an orbital shaker (ELMI DTS-2, Riga, Latvia) with moderate speed (100 rpm) and aliquots of 250 μL of both apical and basolateral compartments were collected after 1 h. All experiments were performed at least in triplicate. Quantification of fluorescence (Na-F: excitation 490 nm, emission 514 nm; LY: excitation 430 nm, emission 535 nm) was carried out using a Chameleon microplate reader (Hidex, Turku, Finland). The apparent permeability coefficient (P_app_) for Na-F and LY was calculated in centimeters/second (cm/s) according to the equation:

$$P_{\mathrm{app}}\left(\mathrm{cm}/s\right)=V_{B}/\left(AC_{A0}\right)\;\times \;\left(\Delta C_{B}/\mathrm{\Delta t}\right),$$

[42] where V_B_ is the volume in the basolateral compartment, A is the surface area of the filter membrane (0.336 cm^2^ for Greiner Bio-one® inserts), C_A0_ is the initial concentration in the apical compartment, and ΔC_B_/Δt is the change of concentration over time in the basolateral compartment.

Recovery (mass balance) of Na-F and LY was calculated with the following equation:

$$\begin{array}{ll} \;\mathrm{Recovery}\;\left(\%\right)= & \left(C_{\mathrm{Af}}V_{A}+C_{\mathrm{Bf}}V_{B}\right)/\left(C_{A0}V_{A}\right)\\ & \;\times \;100\%, \end{array}$$

[43] where C_Af_ and C_Bf_ are the final concentrations of the compound in the apical and basolateral compartment, respectively, C_A0_ is the initial concentration in the apical compartment, and V_A_ and V_B_ are the volumes in the apical and basolateral compartments, respectively. All results are expressed as means ± S.E.M.

## Results

### Biochemical and immunocytochemical characterization of cellular junctions

**Figure 4:**
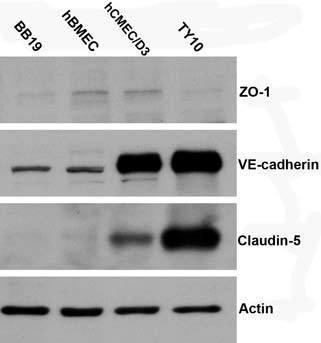
**Western blot analysis of tight junction proteins ZO-1, claudin-5, and adherens junction protein VE-cadherin from cell lysates of four endothelial cell lines.** The cells were lysed with RIPA buffer. 50 μg of total protein was subjected to SDS-PAGE and western blotting analysis for individual markers. Actin was used as loading control. The four cell lines all expressed the adherens junction protein VE-cadherin. The TJ protein ZO-1 was detected in hCMEC/D3, in hBMEC, and at much lower levels in BB19 and TY10 cell lines. Claudin-5 expression was detected in hCMEC/D3, TY10, and at a very low level in hBMEC cells.

**Figure 5:**
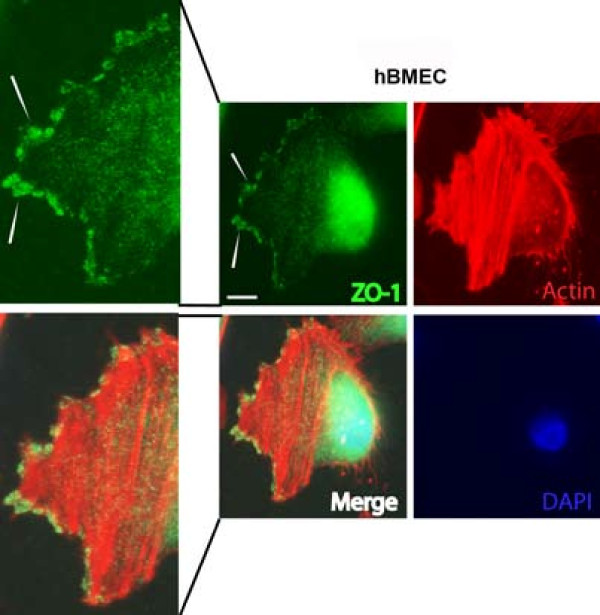
**Immunofluorescence staining of tight junction protein ZO-1 in hBMEC cell line.** hBMEC cells were grown on collagen-coated glass coverslips for 48 h followed by paraformaldehyde fixation. Even with a high background noise level especially on nuclei, ZO-1 was detected at the leading edge of migrating hBMEC cells (see white arrows). Scale bar: 30 μm.

The four endothelial cell lines all expressed the endothelial marker protein VE-cadherin, albeit at different levels (Figure 4). The TJ protein ZO-1 was detected in hCMEC/D3 and in hBMEC at the same level, but it was expressed at much lower levels in BB19 and TY10 cell lines (Figure 4). ZO-1 expression in hBMEC cells was confirmed by immunocytochemical staining (Figure 5). Claudin-5 was not detected in BB19 cells, but was expressed at a high level in TY10 cells, at a low level in hCMEC/D3 cells, and at an even lower level in hBMEC cells (Figure 4).

### Co-culture *in vitro* BBB models

In the beginning of this study, we aimed to establish an all-human *in vitro* BBB model by co-culturing separately several human brain capillary endothelial cell lines (hCMEC/D3, hBMEC, TY10, and BB19) with the human astrocyte cell line SVG-A and with the human pericyte cell line HBPCT. To find the most effective model regarding TJ resistance, each endothelial cell line was grown separately on the apical filter membrane with SVG-A cells or HBPCT cells grown on the basal filter membrane (contact models), or in the culture plate (non-contact models). Various 24-well tissue culture inserts from Corning® with PC and PES membrane and pore sizes 0.4 μm and 3.0 μm were tested, since these insert types are mostly used for drug transport studies.

**Figure 6:**
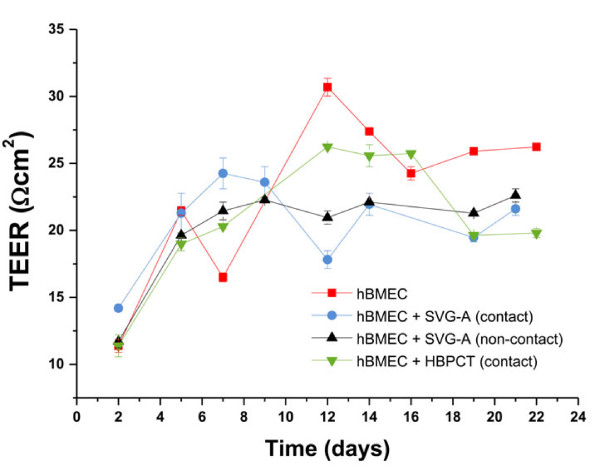
**Mean TEER values for hBMEC cell line in mono-cultures and co-cultures with immortalized astrocytes (SVG-A) and immortalized pericytes (HBPCT).** Experiments using 24-well tissue culture inserts from Corning® (transparent PES membrane, 0.4 μm pore size, 4.0 × 10^6^ pores/cm^2^, n = 2). Maximal TEER values (30.7 ± 0.660 Ωcm^2^ on day 12) were obtained with hBMEC mono-cultures. Co-culture models with SVG-A and HBPCT cells did not result in increased TEER values. Data were recorded with an EVOM coupled to an Endohm-6 measurement chamber.

Maximal TEER values measured with the EVOM were obtained with mono-cultures of hBMEC cells (30.7 ± 0.660 Ωcm^2^ on day 12) on tissue culture insert with PES membrane and 0.4 μm pore size (Figure 6, red curve). hBMEC cells grown in contact or non-contact co-culture models with SVG-A cells on the same kind of inserts resulted in lower maximal TEER values (24.3 ± 1.16 Ωcm^2^ on day 7 and 22.3 ± 0.165 Ωcm^2^ on day 9, respectively) (Figure 6, blue and black curves). In contact co-culture models with HBPCT cells, maximal TEER values were also lower than for hBMEC mono-cultures (26.2 ± 0.165 Ωcm^2^ on day 12 compared to 30.7 ± 0.660 Ωcm^2^ on day 12) (Figure 6, green curve). TEER values for hBMEC cells on additional tested 24-well tissue culture inserts, and TEER values for all other models using hCMEC/D3, TY10, and BB19 cells (in mono-cultures and in co-cultures), yielded maximal TEER values below 20 Ωcm^2^ (data not shown). SVG-A cells and HBPCT cells did not significantly increase or decrease TEER values (data not shown).

### Screening of 24-well tissue culture inserts for each endothelial cell line using the CellZscope

In the above described co-culture experiments, we observed i) that Transwell co-culture models with immortalized astrocytes and immortalized pericytes did not increase TJ resistance of the immortalized brain microvascular endothelial cells included in this study (for hBMEC, see Figure 6), and ii) that membrane material and pore size had a significant impact on barrier tightness. Therefore, we decided to optimize mono-culture models by systematically screening a range of 24-well tissue culture inserts from various providers (Corning®, Greiner Bio-one®, Millipore®, BD Falcon®, Nunc®, and Brand®) for each endothelial cell line, with the aim to increase TEER. For this screening, an automated CellZscope device [30] was used, which records the TEER in real-time every hour inside the incubator at 37°C and 5% CO_2_. TEER values could not be recorded with 24-well tissue culture inserts from Nunc® (PC, 0.4 μm and 3.0 μm pore size) or Brand® (translucent PC, 0.4 μm and 3.0 μm pore size), because their particular design rendered them incompatible with the CellZscope cell module.

### hCMEC/D3 mono-cultures

For hCMEC/D3 monolayers, low TEER values between 5.09 and 11.9 Ωcm^2^ were obtained on all tested inserts (Table 1). It was not possible to identify conclusively the most suitable tissue culture insert for this cell line, since differences between TEER values were not significant. Highest TEER values were observed with 24-well tissue culture inserts from Corning® (transparent PES membrane, 3.0 μm pore size, 2.0 × 10^6^ pores/cm^2^), from Millipore® (translucent PET membrane, 3.0 μm pore size, 2.0 × 10^6^ pores/cm^2^), and from Greiner Bio-one® (transparent PET membrane, 3.0 μm pore size, 0.6 × 10^6^ pores/cm^2^), with a maximum TEER up to 12 Ωcm^2^ (Table 1). For these experiments, hCMEC/D3 cells were cultured with growth medium containing 20% FBS to allow for direct comparisons between all cell lines. When hCMEC/D3 cells were cultured with initial medium containing 5% FBS, maximal TEER values were in the same range (around 10 Ωcm^2^) as when cultured in growth medium containing 20% FBS (Figures 3A and B).

**Table 1:** Summary of maximal TEER values recorded real-time by the CellZscope system for hCMEC/D3, hBMEC, and TY10 mono-cultures

**24-well tissue culture insert coated with collagen type I**	**hCMEC/D3**		**hBMEC**		**TY10**	
	**Time (d)**	**Mean max. TEER ± S.E.M. (Ωcm**^**2**^**)**	**Time (d)**	**Mean max. TEER ± S.E.M. (Ωcm**^**2**^**)**	**Time (d)**	**Mean max. TEER ± S.E.M. (Ωcm**^**2**^**)**
Corning® (translucent PC, 0.4 μm, 1.0 × 10^8^ pores/cm^2^, 0.33 cm^2^)	4.75	6.72 (± 0.113)	2.46	2.79 (± 0.410)	2.37	4.56 (± 1.06)
Corning® (translucent PC, 3.0 μm, 2.0 × 10^6^ pores/cm^2^, 0.33 cm^2^)	5.07	8.56 (± 0.217)	5.19	#9.09 (± 0.103)	7.73**	#11.2 (± 0.904)
Corning® (transparent PES, 0.4 μm, 4.0 × 10^6^ pores/cm^2^, 0.33 cm^2^)	1.97*	7.04 (± 0.231)	2.79*	17.7 (± 0.152)	1.96	6.96 (± 0.199)
Corning® (transparent PES, 3.0 μm, 2.0 × 10^6^ pores/cm^2^, 0.33 cm^2^)	3.38	9.44 (± 0.0877)	2.81	6.24 (± 0.0805)	2.37	7.94 (± 0.110)
Greiner Bio-one® (transparent PET, 0.4 μm, 2.0 × 10^6^ pores/cm^2^, 0.336 cm^2^)	2.47	6.48 (± 0.125)	1.85	#7.27 (± 2.36)	1.45*	9.37 (± 0.690)
Greiner Bio-one® (translucent PET, 0.4 μm, 1.0 × 10^8^ pores/cm^2^, 0.336 cm^2^)	2.34	5.18 (± 0.222)	5.13	7.42 (± 0.288)	1.66*	12.4 (± 2.11)
Greiner Bio-one® (transparent PET, 3.0 μm, 0.6 × 10^6^ pores/cm^2^, 0.336 cm^2^)	2.34	11.9 (± 0.118)	2.75	28.4 (± 2.47)	1.15*	13.0 (± 0.0151)
Greiner Bio-one® (translucent PET, 3.0 μm, 2.0 × 10^6^ pores/cm^2^, 0.336 cm^2^)	2.34	9.39 (± 0.166)	2.49	5.85 (± 0.357)	1.32*	7.26 (± 0.0949)
Millipore® (translucent PET, 0.4 μm, 1.0 × 10^8^ pores/cm^2^, 0.33 cm^2^)	2.34	5.35 (± 0.312)	2.49	2.92 (± 0.164)	1.37*	5.98 (± 0.200)
Millipore® (translucent PET, 3.0 μm, 2.0 × 10^6^ pores/cm^2^, 0.33 cm^2^)	2.34	10.2 (± 0.0304)	2.53	15.0 (± 0.348)	2.37	11.2 (± 0.806)
BD Falcon® (transparent PET, 0.4 μm, 2.0 × 10^6^ pores/cm^2^, 0.3 cm^2^)	2.34	5.09 (± 0.133)	3.34	8.40 (± 0.524)	3.11	4.72 (± 0.00525)
BD Falcon® (transparent PET, 3.0 μm, 0.8 × 10^6^ pores/cm^2^, 0.3 cm^2^)	2.34	8.75 (± 0.180)	3.21	24.1 (± 0.0595)	1.54*	6.19 (± 0.429)

All tested 24-well tissue culture inserts were coated with collagen type I. The cell seeding density varied between 3.0 × 10^4^ and 15 × 10^4^ cells/cm^2^ for hCMEC/D3, between 4.5 × 10^4^ and 17 × 10^4^ cells/cm^2^ for hBMEC, and between 1.5 × 10^4^ and 15 × 10^4^ cells/cm^2^ for TY10 (n = 2–3).

#C_CL_ outside the acceptable range of 0.5-5.0 μF/cm^2^ almost during the whole experiment, indicating that cells were not confluent; PC: polycarbonate; PES: polyester; PET: polyethylene terephthalate; *before first change of medium; **after third change of medium.

### hBMEC mono-cultures

TEER values of hBMEC monolayers were between 2.79 and 28.4 Ωcm^2^. The highest value was measured on 24-well tissue culture inserts (transparent PET membrane, 3.0 μm pore size, 0.6 × 10^6^ pores/cm^2^) from Greiner Bio-one® (Table 1 and Figure 3C).

### TY10 mono-cultures

TEER values for TY10 monolayers were between 4.56 and 13.0 Ωcm^2^. The highest value was recorded when cells were cultured at 37°C from the start of the experiment on inserts from Greiner Bio-one® (transparent PET membrane, 3.0 μm pore size, 0.6 × 10^6^ pores/cm^2^) (Table 1). TEER values were not increased when culturing TY10 monolayers at the permissive temperature of 33°C for 2 days before transferring them to 37°C (Figure 3D).

### BB19 mono-cultures

With monolayers of BB19 cells on various 24-well tissue culture inserts, only extremely low TEER values in the range of 5 Ωcm^2^ were obtained (Figure 3E). Despite repeated experiments, C_CL_ values remained outside of the acceptable range of 0.5-5.0 μF/cm^2^, indicating that cell layers were not confluent. Since barrier restriction with such a low TJ resistance was not sufficient for reliable *in vitro* BBB models, BB19 cell line was not further included in our studies for paracellular tightness assessment using the integrity markers Na-F and LY.

### Further optimization of hBMEC mono-cultures

Since TEER values were highest (28.4 ± 2.47 Ωcm^2^) with monolayers of hBMEC cells cultivated on Greiner Bio-one® inserts (transparent PET membrane, 3.0 μm pore size, 0.6 × 10^6^ pores/cm^2^) coated with collagen type I (Table 1), we optimized this particular mono-culture system by varying parameters such as cell seeding density, coating material and procedure, and by testing different growth media compositions.

Optimization resulted in TEER values in the range of 40 Ωcm^2^ (Table 2). A maximal TEER value of 43.6 ± 3.89 Ωcm^2^ (after 3.19 days *in vitro*) was obtained when hBMEC cells were cultured under normal growth conditions (growth medium containing 20% FBS) at a seeding density of 4.5 × 10^4^ cells/cm^2^ (Table 2). Lower or higher cell seeding densities resulted in decreased maximal TEER values (25.9 ± 0.635 Ωcm^2^ at 3.0 × 10^4^ cells/cm^2^ and 28.4 ± 2.47 Ωcm^2^ at 15 × 10^4^ cells/cm^2^). The duration of coating the tissue culture inserts with collagen type I did not have an impact on TEER (data not shown). Coating the inserts with collagen type IV/fibronectin did not further improve barrier tightness. However, coating the tissue culture inserts with matrigel resulted in a decrease of maximal TEER values (16.6 ± 0.183 Ωcm^2^ compared to 28.4 ± 2.47 Ωcm^2^). Growth media containing barrier inducing compounds such as dexamethasone (500 nM and 1000 nM, respectively), 8-(4-CPT)-cAMP (250 μM), RO-20-1724 (17.5 μM), additional HC (500 nM and 1500 nM, respectively), or additional hEGF (50 ng/mL and 100 ng/mL, respectively) did not significantly increase the TEER of hBMEC monolayers. Growth medium supplemented with HS (10% and 20%, respectively) instead of 20% FBS resulted in a decreased maximal TEER (20.4 ± 0.867 Ωcm^2^ and 26.2 ± 0.324 Ωcm^2^, respectively, compared to 28.4 ± 2.47 Ωcm^2^). The use of ACM-1 resulted in a decrease of the maximal TEER (13.5 ± 0.614 Ωcm^2^, compared to 28.4 ± 2.47 Ωcm^2^). A similar result, i.e. a decrease of maximal TEER, was obtained when using a mixture of ACM-2 and growth medium 20% FBS (1:1) (23.8 ± 0.572 Ωcm^2^, compared to 28.4 ± 2.47 Ωcm^2^).

**Table 2:** Summary of maximal TEER values recorded real-time by the CellZscope system for hBMEC mono-cultures using a range of different culture conditions

**Culture conditions**	**Cell seeding density (cells/cm**^**2**^**)**	**Time (d)**	**Mean max. TEER ± S.E.M. (Ωcm**^**2**^**)**
Cell seeding density: 3.0 × 10^4^ cells/cm^2^, coating: collagen type I (10 μg/cm^2^)	3.0 × 10^4^	1.72*	25.9 (± 0.635)
Cell seeding density: 4.5 × 10^4^ cells/cm^2^, coating: collagen type I (10 μg/cm^2^)	4.5 × 10^4^	3.19	43.6 (± 3.89)
Growth medium with additional HC (500 nM) for 3 days (only apical), coating: collagen type I (10 μg/cm^2^)	4.5 × 10^4^	3.19	43.2 (± 4.27)
Growth medium with additional HC (1500 nM) for 3 days (only apical), coating: collagen type I (10 μg/cm^2^)	4.5 × 10^4^	3.19	40.4 (± 0.646)
Cell seeding density: 6.0 × 10^4^ cells/cm^2^, coating: collagen type I (10 μg/cm^2^)	6.0 × 10^4^	1.72*	36.3 (± 2.06)
Cell seeding density: 9.0 × 10^4^ cells/cm^2^, coating: collagen type I (10 μg/cm^2^)	9.0 × 10^4^	4.15	38.9 (± 0.928)
Growth medium with dexamethasone (500 nM) for 3 days (only apical), coating: collagen type I (10 μg/cm^2^)	9.0 × 10^4^	4.15	35.9 (± 0.338)
Growth medium with dexamethasone (1000 nM) for 3 days (only apical), coating: collagen type I (10 μg/cm^2^)	9.0 × 10^4^	4.15	38.0 (± 1.26)
Growth medium with additional hEGF (50 ng/mL) for 3 days (only apical), coating: collagen type I (10 μg/cm^2^)	9.0 × 10^4^	4.15	36.4 (± 0.902)
Growth medium with additional hEGF (100 ng/mL) for 3 days (only apical), coating: collagen type I (10 μg/cm^2^)	9.0 × 10^4^	4.15	40.0 (± 1.94)
Growth medium with 8-(4-CPT)cAMP (250 μM) and RO-20-1724 (17.5 μM) (apical and basolateral), coating: collagen type I (10 μg/cm^2^)	9.0 × 10^4^	2.84	20.0 (± 0.509)
Growth medium with 8-(4-CPT)cAMP (250 μM) and RO-20-1724 (17.5 μM), added after 2 days *in vitro* (apical and basolateral), coating: collagen type I (10 μg/cm^2^)	9.0 × 10^4^	2.84	20.9 (± 1.36)
Cell seeding density: 15 × 10^4^ cells/cm^2^, coating: collagen type I (10 μg/cm^2^)	15 × 10^4^	2.75	28.4 (± 2.47)
Coating: collagen type IV/fibronectin (80/20 μg/cm^2^)	15 × 10^4^	2.75	29.3 (± 1.10)
Coating: matrigel (80 μg/cm^2^)	15 × 10^4^	2.75	16.6 (± 0.183)
Growth medium with 10% HS (apical and basolateral), coating: collagen type I (10 μg/cm^2^)	15 × 10^4^	1.26*	20.4 (± 0.867)
Growth medium with 20% HS (apical and basolateral), coating: collagen type I (10 μg/cm^2^)	15 × 10^4^	1.21*	26.2 (± 0.324)
ACM-1 (apical and basolateral), coating: collagen type I (10 μg/cm^2^)	15 × 10^4^	1.64*	13.5 (± 0.614)
Mixture of ACM-2 and growth medium 20% FBS (1:1) (apical and basolateral), coating: collagen type I (10 μg/cm^2^)	15 × 10^4^	3.35	23.8 (± 0.572)

24-Well tissue culture inserts were from Greiner Bio-one® (transparent PET membrane, pore size 3.0 μm, 0.6 × 10^6^ pores/cm^2^, n = 2–3). *Before first change of medium.

### Evaluation of paracellular permeability through mono-cultures

**Figure 7:**
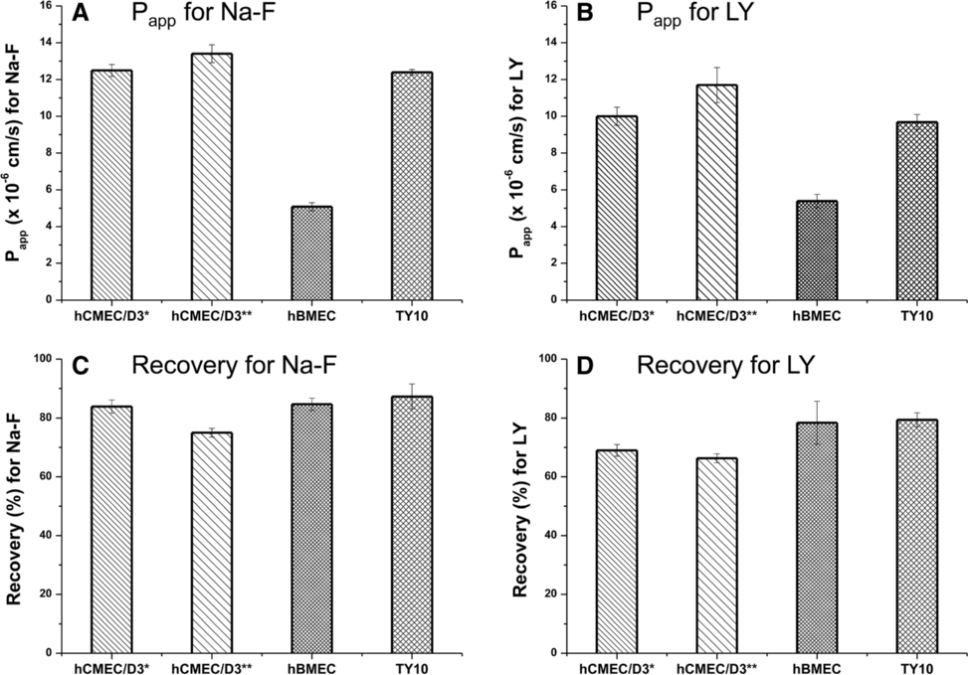
**Mean P**_**app **_**values (A, B) and mean recoveries (C, D) for Na-F and LY through hCMEC/D3, hBMEC, and TY10 monolayers.** 24-Well tissue culture inserts from Greiner Bio-one® (transparent PET membrane, 3.0 μm pore size, 0.6 × 10^6^ pores/cm^2^) were used (n = 3–5). **A**, **B**: Lowest paracellular permeability of Na-F and LY was obtained through hBMEC monolayers (5.08 ± 0.220 × 10^-6^ cm/s and 5.39 ± 0.364 × 10^-6^ cm/s, respectively). All experiments for Na-F permeability were carried out at the time indicated with a black arrow in Figures 3A-D. For LY, permeability measurements were carried out at TEER values in the same range (graphs not shown). **C**, **D**: Mean recoveries of Na-F in all experiments were in the range of 75% and 87%. For LY, mean recoveries varied between 66% and 79%. *cultured with initial medium; **cultured with growth medium 20% FBS.

Since highest TEER values were observed when hBMEC monolayers were cultured on rat-tail collagen type I coated Greiner Bio-one® inserts (transparent PET membrane, pore size 3.0 μm, 0.6 × 10^6^ pores/cm^2^) at a cell seeding density of 4.5 × 10^4^ cells/cm^2^ (Table 1), this insert was selected for permeability studies using Na-F (MW 376.27) and LY (MW 550.39). For hBMEC cell line, the mean P_app_ for Na-F was 5.08 ± 0.220 × 10^-6^ cm/s (Figure 7A). For LY, the mean P_app_ was 5.39 ± 0.364 × 10^-6^ cm/s (Figure 7B). For hCMEC/D3, mean P_app_ values for Na-F and LY were 12.5 ± 0.326 × 10^-6^ cm/s and 10.0 ± 0.498 × 10^-6^ cm/s, respectively, when cells were grown with initial medium containing 5% FBS (Figures 7A and B). When hCMEC/D3 cells were cultivated with growth medium containing 20% FBS, mean P_app_ values for Na-F and LY were 13.4 ± 0.484 × 10^-6^ cm/s and 11.7 ± 0.957 × 10^-6^ cm/s, respectively (Figures 7A and B). For TY10, mean P_app_ values for Na-F and LY were 12.4 ± 0.155 × 10^-6^ cm/s and 9.68 ± 0.413 × 10^-6^ cm/s, respectively (Figures 7A and B). Mean recoveries for Na-F in all experiments were between 75% and 87% (Figure 7C). For LY, mean recoveries were between 66% and 79% (Figure 7D). According to Hubatsch et al. (2007), a mass balance of at least 80% would be optimal to give an acceptable approximation of the P_app_ value [44]. After each experiment, TEER values were in the same range as before the assay (or even higher) (Figures 3A-D), suggesting that cell monolayers were robust during the experiment and barrier integrity was maintained.

### Evaluation of paracellular permeability through contact co-cultures

**Figure 8:**
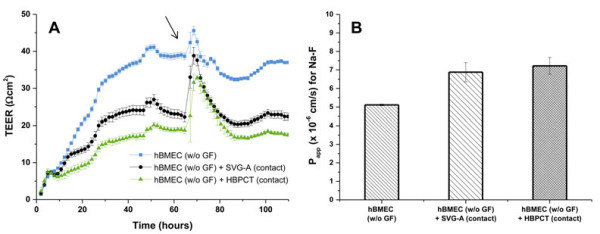
**Mean TEER (A) and P**_**app **_**(B) values for hBMEC cultured without growth factors in mono-cultures and contact co-cultures with SVG-A and HBPCT cells. A**: Mean TEER values recorded real-time by the CellZscope system for hBMEC cells cultured without growth factors (w/o GF) in mono-cultures and co-cultures with SVG-A (black curve) or HBPCT cells (green curve). 24-Well tissue culture inserts from Greiner Bio-one® (transparent PET membrane, 3.0 μm pore size, 0.6 × 10^6^ pores/cm^2^) were used (n = 3). Compared to co-cultures with SVG-A and HBPCT cells, hBMEC mono-cultures produced higher TEER values in the range of 40 Ωcm^2^ (blue curve). C_CL_ values were in the acceptable range of 0.5-5.0 μF/cm^2^ (data not shown), indicating that hBMEC cells were confluent. The black arrow indicates the time when the permeability assay with Na-F was performed. **B**: Mean P_app_ values for Na-F through hBMEC cultured without growth factors through mono-cultures and co-cultures with SVG-A or HBPCT cells. Lowest P_app_ values for Na-F (5.11 ± 0.0487 × 10^-6^ cm/s) were obtained through hBMEC mono-cultures. Mean recoveries in all experiments were between 98% and 102% (n = 3).

Since lowest P_app_ values were obtained with hBMEC monolayers (Figures 7A and B), we established co-culture models with SVG-A and HBPCT cells to further assess paracellular permeability. Mean P_app_ values for Na-F through hBMEC co-cultured with SVG-A and HBPCT cells were 7.43 ± 0.200 × 10^-6^ cm/s and 8.26 ± 0.893 × 10^-6^ cm/s, respectively (compared to 5.08 ± 0.220 × 10^-6^ cm/s through hBMEC mono-cultures). Since a culture medium supplemented with growth factors may mask the potential BBB inducing effect of astrocytes and pericytes, further experiments were performed using culture medium without growth factors. Mean P_app_ values for Na-F through hBMEC monolayers cultured without growth factors was 5.11 ± 0.0487 × 10^-6^ cm/s (Figure 8). Through hBMEC cells co-cultured with SVG-A or HBPCT cells using medium deprived of growth factors, mean P_app_ values were slightly higher (6.88 ± 0.516 × 10^-6^ cm/s and 7.22 ± 0.455 × 10^-6^ cm/s, respectively) (Figure 8). In all experiments, mean recoveries were between 98% and 102%.

## Discussion

There is a need for predictive assays amenable to medium to high-throughput screening for the assessment of brain penetration of drug leads. To this date, only a few immortalized human brain capillary endothelial cell lines have been developed and used for the establishment of human *in vitro* BBB models [9]. The most extensively characterized human cell line is the hCMEC/D3 cell line, which has been reported to represent a promising *in vitro* human BBB model for drug transport studies [45,46]. TY10 cell line, transduced with a temperature-sensitive SV40 large-T antigen, has furthermore been reported to be a promising and advantageous cell line with excellent expression of TJ proteins such as claudin-5, occludin, and ZO-1 [26]. Whereas at 33°C, TY10 cells can be cultivated for more than 50 passages without undergoing morphological changes, a temperature shift from 33°C to 37°C results in the exclusion of the SV40 large-T antigen as a cancer gene [33,34].

However, monolayers of immortalized human brain capillary endothelial cell lines are known to form only moderately restrictive barriers, with TEER values in the range of 20–200 Ωcm^2^[17,20,21]. To increase barrier tightness of four currently available human brain capillary endothelial cell lines, hCMEC/D3, hBMEC, TY10, and BB19 (Figure 1), we tested co-culture models with immortalized human astrocytes (SVG-A cell line) and pericytes (HBPCT cell line). Interestingly, we did not observe an increase of TJ resistance in immortalized human brain capillary endothelial cells under co-culture conditions. All co-culture models produced TEER values that were comparable or lower than those recorded with mono-cultures (for hBMEC cell line, see Figures 6 and 8A). This suggested that the investigated endothelial cell lines were unable to respond positively to stimuli from immortalized astrocytic or pericytic cells.

These findings are in accord with previous reports in which no additional benefit in terms of TEER was observed when culturing hCMEC/D3 cells with ACM or when co-culturing the endothelial cells with human astrocytes, respectively [24,47]. In contrast, our results do not support recent findings which showed that TJ resistance of hCMEC/D3 and TY08 cells was significantly increased in co-cultures with human brain astrocytes/pericytes and HBPCT cells, respectively [29,48]. This divergent effect might possibly be due to the difference of culture conditions and the nature of cell types.

We observed that membrane material of inserts and pore size had a significant impact on barrier tightness, and we optimized mono-culture systems by systematically screening a large set of 24-well tissue culture inserts from different providers for each cell line (Table 1). Highest TEER values (28.4 ± 2.47 Ωcm^2^) were observed with hBMEC mono-cultures on 24-well tissue culture inserts from Greiner Bio-one® with transparent PET membrane and 3.0 μm pore size (Table 1). Our findings clearly show that the selection of an appropriate tissue culture insert is critical when establishing a BBB model using these immortalized human brain capillary endothelial cell lines, corroborating previous findings in which a substantial impact of material characteristics on the adherence of cells and barrier tightness was demonstrated [31,32]. hCMEC/D3 and TY10 cells produced TEER values in the range of 10 Ωcm^2^ (Table 1). BB19 cells were not included into the screening of tissue culture inserts, since these cells yielded extremely low TEER values (around 5 Ωcm^2^, Figure 3E). These findings are in agreement with previous studies suggesting that the use of BB19 cells as an *in vitro* model of the human BBB is limited due to a high sucrose permeability [49].

Surprisingly, we consistently observed lower TEER values as compared to literature (hCMEC/D3 and hBMEC: TEER ranging from 40–200 Ωcm^2^[21,24,25], TY10: TEER in the range of 40 Ωcm^2^[26]). A possible explanation may be that we used a different system (automated CellZscope) for assessment of TEER [30]. Low TEER values might also arise from a high concentration of serum and growth factors in the growth medium, which has been reported to prevent TJ formation between endothelial cells [50]. However, hCMEC/D3 cells cultured with growth medium containing 5% FBS instead of 20% FBS, and hBMEC cells cultured in growth medium without growth factor supplementation did not result in increased TEER values or reduced paracellular permeability (for hCMEC/D3, see Figures 3A, 3B, Figures 7A, and 7B, for hBMEC see Figure 8). Furthermore, the selection of the well format might affect TEER values. Because we aimed to establish an *in vitro* BBB model suitable for higher throughput, we miniaturized the assay to a 24-well format that was selected previously for a bovine *in vitro* BBB model [41].

Subsequent optimization of the hBMEC mono-culture system resulted in TEER values in the range of 30–40 Ωcm^2^ (Table 2). We found that the cell seeding density is critical, since highest TEER values were obtained when hBMEC cells were seeded at a density ranging between 4.5 × 10^4^ and 9.0 × 10^4^ cells/cm^2^ onto coated inserts (Table 2). These conditions seem to be best for cell growth and TJ development between adjacent cells on the surface area of the Greiner Bio-one® inserts (0.336 cm^2^). As hBMEC cells are of human origin, we moreover investigated the effect of HS on barrier tightness. However, no increase in TEER could be observed (Table 2). These results do not confirm previous findings that the permeability of sucrose through hCMEC/D3 monolayers could significantly be reduced by HS supplementation [45]. One may speculate that this could be due to a different type of HS used in these experiments. An individual HS batch as used for our experiments is prone to higher batch-to-batch variation in soluble factors and proteins than pooled HS from commercial sources.

In the evaluation of paracellular permeability, mean P_app_ values for Na-F and LY were significantly lower with hBMEC monolayers (5.08 ± 0.220 × 10^-6^ cm/s and 5.39 ± 0.364 × 10^-6^ cm/s, respectively) than with hCMEC/D3 and TY10 monolayers (Figures 7A and B), corroborating our measurements of TEER values. Permeability values in the order of 10^-6^ cm/s were obtained previously in various *in vitro* BBB models [7,20,39,51].

In the biochemical and immunocytochemical characterization of cellular junctions, VE-cadherin was detected in all cell lines, albeit at varying levels (Figure 4). This confirmed their endothelial lineage. Interestingly, the TJ protein claudin-5 was expressed at similar levels as VE-cadherin, confirming that VE-cadherin controls claudin-5 expression [52]. The cellular junction marker protein ZO-1 showed the same level of expression in hCMEC/D3 and hBMEC cells, but was expressed only at very low levels in BB19 and TY10 cells (Figure 4). Furthermore, white arrows in Figure 5 point to ZO-1 signal at the leading edge of migrating hBMEC cells, confirming findings of previous studies [13].

We used an automated CellZscope system [30] in order to obtain highly standardized data on-line. This system has several advantages over other methods of TEER measurement: TEER values are recorded in real-time every hour in the incubator, thereby reducing workload and avoiding any damage of the cell layer during growth. Also, a disruption of the cell layer is immediately visible from the recording. In addition, information about confluency (C_CL_ values) is obtained simultaneously, reducing the risk of false interpretation of TEER values [53]. Experiments with Caco-2 cells on 24-well inserts demonstrated that the CellZscope is likewise an efficient tool for evaluating barrier tightness in other cell lines, i.e. those used for the study of intestinal drug absorption. Again, independent of the membrane surface area, TEER values of Caco-2 monolayers measured with the CellZscope (24-well format) correlated to off-line TEER values measured manually with an EVOM using a 6-well format (data not shown). One limitation of the CellZscope system may be its design which does not allow the seeding of cells on the bottom of the plate. Investigation of triple co-culture model systems is hence not possible.

## Conclusions

In the screening of four available immortalized human brain capillary endothelial cell lines, hBMEC proved to be the most suitable and promising cell line for a human *in vitro* BBB model in terms of barrier tightness and paracellular permeability in a 24-well mono-culture system. hBMEC cells express P-gp [54], claudin-1 [55], claudin-3 [56], occludin [55-57], ZO-1 [54-56,58], ß-catenin [58], ICAM-1 [56], and VCAM-1 [56], some of which were also shown under our experimental conditions (VE-cadherin, ZO-1, and claudin-5, see Figures 4 and 5). Interestingly, although all three examined markers were detected in hBMEC, the expression level of VE-cadherin and claudin-5 was much lower than in hCMEC/D3 and TY10 cells. As a next step, we are currently validating the hBMEC model with the aid of a series of compounds known to cross or not to cross the BBB. After validation, the *in vitro* human BBB model will be used for the screening of natural product derived leads, such as GABA_A_ receptor modulators [59], regarding their ability to pass across the BBB.

## Abbreviations

ACM: Astrocyte-conditioned medium; BB19: Immortalized human brain capillary endothelial cells; BBB: Blood–brain barrier; CCL: Cell layer capacitance; CNS: Central nervous system; EVOM: Epithelial voltohmmeter; FBS: Fetal bovine serum; hBMEC: Immortalized human brain microvascular endothelial cell line; HBPCT: Human brain pericyte cell line; HC: Hydrocortisone; hCMEC/D3: Immortalized human cerebral microvascular endothelial cell line D3; hEGF: Human epidermal growth factor; HS: Human serum; LY: Lucifer yellow VS dilithium salt; Na-F: Sodium fluorescein; NCEs: New chemical entities; Papp: Apparent permeability coefficient; PC: Polycarbonate; PES: Polyester; PET: Polyethylene terephthalate; P-gp: P-glycoprotein; RIPA: Standard radio-immunoprecipitation assay; S.E.M.: Standard error of the mean; SVG-A: Human astrocyte cell line; TEER: Transendothelial electrical resistance; TJ: Tight junction; TY10: Conditionally immortalized human brain microvascular endothelial cell line; VE-cadherin: Vascular endothelial-cadherin; ZO-1: Zonula occludens-1 protein.

## Competing interests

The authors declare that they have no competing interests.

## Authors’ contributions

DEE, MO, and GX designed and performed the experiments, analyzed the data and drafted the manuscript. DEE, MO, GX, KSK, AVM, and MH edited and critically reviewed the manuscript. KSK and AVM kindly supplied cells. All authors read and approved the final manuscript.
